# A Fissure-Aided Registration Approach for Automatic Pulmonary Lobe Segmentation Using Deep Learning

**DOI:** 10.3390/s22218560

**Published:** 2022-11-07

**Authors:** Mengfan Xue, Lu Han, Yiran Song, Fan Rao, Dongliang Peng

**Affiliations:** 1 School of Automation, Hangzhou Dianzi University, Hangzhou 310018, China; 2 Research Center for Healthcare Data Science, Zhejiang Lab, Hangzhou 311121, China; 3 Philips Healthcare, Shanghai 200072, China

**Keywords:** medical imaging, image processing, segmentation

## Abstract

The segmentation of pulmonary lobes is important in clinical assessment, lesion location, and surgical planning. Automatic lobe segmentation is challenging, mainly due to the incomplete fissures or the morphological variation resulting from lung disease. In this work, we propose a learning-based approach that incorporates information from the local fissures, the whole lung, and priori pulmonary anatomy knowledge to separate the lobes robustly and accurately. The prior pulmonary atlas is registered to the test CT images with the aid of the detected fissures. The result of the lobe segmentation is obtained by mapping the deformation function on the lobes-annotated atlas. The proposed method is evaluated in a custom dataset with COPD. Twenty-four CT scans randomly selected from the custom dataset were segmented manually and are available to the public. The experiments showed that the average dice coefficients were 0.95, 0.90, 0.97, 0.97, and 0.97, respectively, for the right upper, right middle, right lower, left upper, and left lower lobes. Moreover, the comparison of the performance with a former learning-based segmentation approach suggests that the presented method could achieve comparable segmentation accuracy and behave more robustly in cases with morphological specificity.

## 1. Introduction

Human lungs are generally divided into five lobes. The pulmonary fissure is an important anatomic landmark of the lung. The right lung is separated into an upper lobe, middle lobe, and lower lobe by the horizontal fissure and oblique fissure, respectively, while the left lung is separated into an upper lobe and lower lobe by the oblique fissure. Each lobe is supplied by its own bronchial tree, which is subdivided from the main bronchi. Vascular, lymphatic vessels, and autonomic nerves in each lobe are also largely isolated. Additionally, the influence of gravity leads to differences in ventilation and perfusion in these relatively independent functional lobes. Therefore, there are several types of lung diseases that affect the pulmonary lobes differently. Generally, pulmonary fibrosis and tuberculosis predominantly involve the upper lobe [1]. Pulmonary edema and interstitial pneumonias have a lower lobe predominance [2]. As for the right middle lobe, the syndrome occurs frequently [3]. Hence, segmentation of the pulmonary lobes based on CT scans is important in clinical practice. It contributes to physiological status assessment, lesion location, and surgical planning. For example, pre-interventional lobe segmentation and quantitative evaluation facilitate the treatment planning of lung volume reduction (LVR), which is an invasive therapy for emphysema [4].

In today’s human living conditions, environmental pollution is getting worse and worse. Diseases, such as those of the lung and respiratory tract, have become the main factors leading to premature human deaths due to their high incidence rate and mortality. Computed tomography (CT) is the standard modality to detect, diagnose, and prognosticate the lung diseases in clinical settings. CT images could provide high spatial resolution and very detailed anatomical information. CT is currently the most sensitive imaging modality for detecting the fine structure of the lungs, and it is also the preferred modality for detecting pulmonary pathology. Moreover, the scan is rapid and noninvasive. 

Although an experienced radiologist is able to segment the lung lobes by tracing the pulmonary fissures from the CT scans, the task is rarely performed in clinical practice. Manual segmentation is time-consuming and labor-intensive due to the large number of images. Moreover, the result of segmentation is prone to subjective bias and variance. Thus, there is a need for a robust automatic lobe segmentation scheme in clinical practice. 

Over the past decades, many authors have proposed methods for pulmonary lobe segmentation from CT scans [5,6,7]. Since the task of pulmonary lobe segmentation is not difficult to achieve if the fissures can be accurately delineated, early methods show great interest in fissure detection [8,9,10,11]. Most of these methods are based on the anatomical characteristics of the pulmonary fissure, which is represented as a curved line in a 2D image space and a thin planar structure in a 3D image space.

However, studies have shown that the fissures are frequently incomplete, making lobe segmentation a challenging task. Methods vary according to how the incomplete problem is handled. As radiologists utilize information from the bronchi and vascular trees when inferring the incomplete fissures, some methods propose to segment other auxiliary structures as cues for the lobe border determination. Kuhnigk et al. [12] made use of the absence of major vessels at the lobar boundaries. They calculated the Euclidean distance from the nearest vessel for each voxel to quantify the absence. The resulting distance map and original density map constructed a cost map, guiding the watershed algorithm to separate the lobes. Lassen et al. [13] improved the fissure accuracy by extending the work of Kuhnigk et al. [12]. The extended method took additional lobar fissures and airway information into consideration when constructing the cost image and is shown to outperform the method by Kuhnigk et al. Different from Lassen et al., who directly included detected fissures in the watershed segmentation [13], Ukil and Reinhardt [14] treated the resulting watershed basins as a region of interest, incorporating the fissures. They also developed an incomplete fissure detection method, using a 3D graph search to extrapolate the optimal fissure surface.

Other kinds of methods indirectly segment the lung lobe by registering the image to be segmented with the atlas annotated with lung lobes. Zhang et al. [15] constructed a pulmonary atlas and deformed it to match the target data for coarse initialization of the fissure surfaces [16]. Then fissure surfaces were refined by a fuzzy reasoning system based on the ridgeness map, the anatomic smoothness constraint, and the fissure initialization [17]. Van Rikxoort et al. [18] introduced a multi-atlas-based registration to overcome the deformation limitations due to the large anatomical variation in the shape of lobes among different subjects. They emphasized the lobar borders and the fissures in the registration. Information from the bronchial trees was only used where fissures were incomplete to guide the registration.

Recently, deep learning methods have been rapidly applied in the field of medical image processing and analysis [19]. U-Net [20], consisting of a contracting path and a symmetric expanding path, outperformed many previous deep network approaches in 2D medical image segmentation. Milletari et al. [21] extended the U-Net architecture to 3D scenarios, called the V-Net. Ferreira et al. [22] added advanced regularization techniques to the V-Net and used the resulting model, the FRV-Net, in lobe segmentation to overcome the lack of annotated training data. Deep learning methods were also explored to address the difficulties encountered in traditional lung lobe segmentation algorithms. Gerard et al. [23] designed the FissureNet, a coarse-to-fine cascade of two SegNet [24] for thin structure segmentation. It made good use of local structure and contextual information to accurately segment the pulmonary fissures.

In this work, we propose an automatic lobe segmentation method that incorporates knowledge from the local pulmonary boundaries, global context, and priori pulmonary anatomy to separate the lobes robustly and accurately. It applies a learning-based registration approach to fuse local fissure and lung context information. A comparison with FRV-Net, a learning-based segmentation method, is created to evaluate the performance of the proposed method. We also release our 24 CT scans and corresponding manual annotations as a reference for further study.

## 2. Method

**Figure 1:**
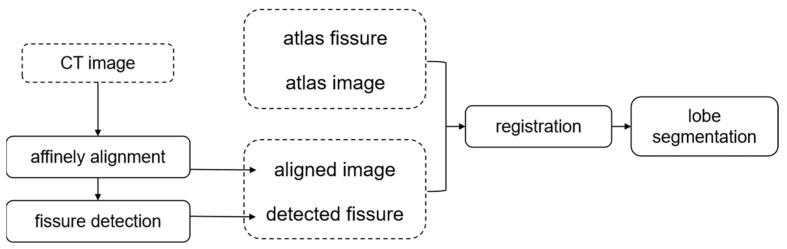
Overview of the fissure-aided lung registration method for lobe segmentation.

An automatic lobe segmentation method is developed employing the fissures, the lungs, and the atlas-based guidance. Figure 1 presents an overview of our method. The process starts by affinely aligning the test CT images to the atlas, so that the remaining source resulting in misalignment is nonrigid. Then, the fissures are segmented from the aligned CT images (Section 2.1). Directly registering the aligned CT images and the atlas can only yield an acceptable match of the lung volume. A pulmonary fissure is helpful for the localization of pulmonary lobes. The important role that the fissure plays in the description of the lobe specificity cannot be ignored. Hence, in this work, the prior lobe knowledge is introduced to infer the lobar border against the incomplete fissure by registering the aligned CT images and the atlas together with their fissures.

**Figure 2:**
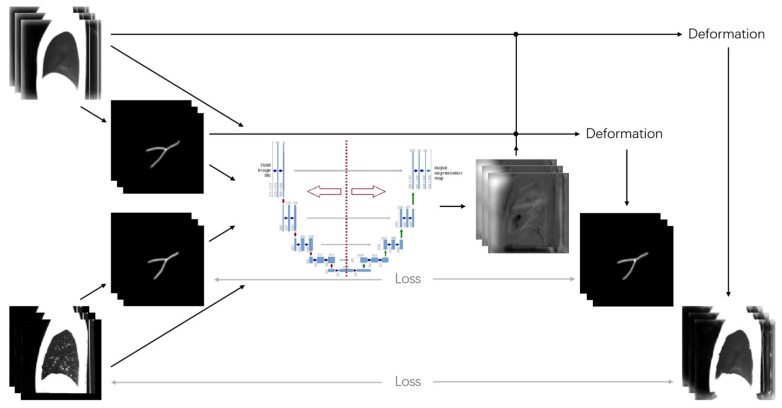
Illustration of the fissure-based registration method.

A learning-based registration method is used for the nonrigid transformation (see Figure 2), which is accurate and operates faster. The lobe segmentation of the test CT images is implemented by mapping the deformation field information generated from the registration step to the lobe-annotated atlas. Additionally, the final result of the lobe segmentation is obtained through a post-processing step to further classify the voxels with unreasonable deformation after mapping.

### 2.1. Prerequisite Segmentations

(1) Lung Segmentation

Lung segmentation is a preprocessing step for subsequent lung fissure extraction. The lung is segmented with an automatic method based on previous work [25]. Briefly, the method includes three steps: airspace segmentation, bronchial separation, and lung closure.

First, a 3D fixed-threshold region-growing algorithm is performed to segment the pulmonary airspace. The two seed points from the left and right lung are detected by scanning for the airspace points around the chosen fixed points in the affinely registered CT images. Second, the bronchial tree is separated by using an optimal threshold region that grows with seeds in the main stem bronchi and is automatically detected from top to bottom. Last, a morphological closing operation is applied in the remaining airspaces to close pulmonary vessels and pathological changes.

(2) Fissure Segmentation

The pulmonary fissure is segmented according to its bright, thin sheet-like feature in 3D CT images [13,26]. Since the relationship among eigenvalues in the Hessian matrix discriminates the sheet, tube, and blob structures, the pulmonary fissure is first enhanced by calculating a sheet similarity derived from the Hessian matrix for each voxel to filter the candidate fissure voxels. As for a local sheet-like fissure, one eigenvalue is large ($\left|\lambda_{3,i}\right|\gg 0$) and the other two eigenvalues are small ($\left|\lambda_{1,i}\right|,\left|\lambda_{2,i}\right|\approx 0$). Lassen et al. [13] introduced structure and sheet feature functions and took them into a fissure similarity model $S_{fissure,i}$ defined as follows:

(1) $$S_{fissure,i}=F_{structure,i}F_{sheet,i}$$

(2) $$F_{structure,i}=\Theta \left(-\lambda_{3,i}\right)e^{-\frac{{\left(\lambda_{3,i}-\alpha \right)}^{6}}{\beta^{6}}}\;,\;\Theta \left(-\lambda_{3,i}\right)=\{\begin{array}{c} \;0,\;\lambda_{3,i}\geq 0\;\\ 1,\;\lambda_{3,i}<0 \end{array}$$

(3) $$F_{sheet,i}=e^{-\lambda_{2,i}{}^{6}/\gamma^{6}}$$

where $F_{structure,i}$ rates the gray scale in the ith voxel, $F_{sheet,i}$ rates the sheet structure possibility for the ith voxel. This model defines high fissure probability for the bright sheet structure on a dark background. Empirically, α is set to 50; β is set to 35; γ is set to 25; and a voxel with $S_{fissure,i}>0.1$ is considered a potential fissure voxel.

**Figure 3:**
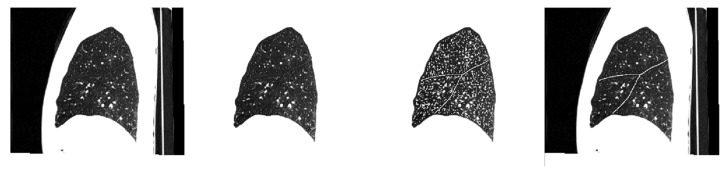
Illustration of fissure segmentation.

Then, a three-dimensionally connected component analysis based on the inner product of the orientation eigenvectors from two neighboring voxels is applied to extract the continuous fissures from the potential fissure voxels. The largest eigenvalue of a sheet structure of the Hessian matrix corresponds to the eigenvectors directing the plane. As the pulmonary fissures are smooth and flat, two adjacent potential fissure voxels are labeled as the same connected component, if their directions are similar (inner product ≥ 0.95). Finally, connected components with a number of counts greater than a fixed value are filtered as the fissure segmentation result. In all our instances, we successfully identified single connected components as fissures. An example of fissure segmentation is shown in Figure 3.

The first frame shows the original sagittal slice. In the second frame the corresponding segmented lung is shown. The third frame shows the result of fissure enhancement by calculating the fissure similarity. The last frame shows the extracted fissure on the original slice.

### 2.2. Learning-Based Registration

Compared with traditional nonlearning-based registration methods that optimize the objective function for each image pair, learning-based approaches treat registration as a mapping function from the fixed and moving images to the deformation field. Balakrishnan et al. proposed VoxelMorph [27,28], an unsupervised learning-based registration method that could rapidly predict a deformation field by evaluating the learned global function.

VoxelMorph has an encoder-decoder structure and skips the connections added between the down-sampling and up-sampling parts, which is similar to the classical U-net [20,29] architecture. VoxelMorph uses spatial transformer networks (STN) [30] to produce moved images (m∘ϕ), and the loss function is designed to maximize the similarity between the fixed image (f) and the moved image. Furthermore, smoothness regularization is introduced to penalize irregular local variations of the deformation field (ϕ), and  λ is a parameter that adjusts the strength of the penalty term.

Spatial transformer networks have three parts, which are respectively responsible for parameter prediction, coordinate mapping, and pixel acquisition. The mapping relationship is from the target image to the original image. Here, it refers to moving from the fixed image (f) to the moving image (m). The function of coordinate mapping is actually to sample the target image of the original image, collect pixels from different coordinates of the original image to the target image every time, and paste the target image fully. The coordinates of the target image must be traversed every time, and are fixed, while the coordinates of the collected original image are not fixed.

Only the input volume and the generated registration field were used to evaluate the unsupervised loss of the model. The unsupervised loss $L_{VM}$ consists of two components: $L_{similarity}$ that penalizes differences in appearance, and $L_{smooth}$ that penalizes local spatial variations in φ:

(4) $$L_{VM}=L_{similarity}\left(f,m\circ \phi \right)+\lambda L_{smooth}\left(\phi \right)$$

where *λ* is a regularization parameter.

### 2.3. Pulmonary Lobe Segmentation

In this experiment, we train VoxelMorph for atlas-based registration. Through our preliminary experiments, we found that direct application of VoxelMorph to chest CT scans to achieve lobe segmentation does not work well. Although the chest CT lung volume could precisely match the atlas lung volume, which provides useful information about morphological constraints for lobe segmentation, the fissures are not deformed to the corresponding positions.

In this study, we propose a fissure-aided lung registration method to segment pulmonary lobes. We combined the affine-aligned CT images and the extracted fissure images, and the same method was also used in the atlas images and the atlas fissure. Then, these two sets of images are used as the input of VoxelMorph, that is, one is the moving image (m) and the other is the fixed image (f). To enhance the robustness of the fissure-aided registration method, the similarity of fissures is given equal weight to that of the lungs. The loss function is defined as follows:

(5) $$L_{LS}=L_{similarity,CT}\left(f_{CT},m_{CT}\circ \phi \right)+L_{similarity,f}\left(f_{f},m_{f}\circ \phi \right)+\lambda L_{smooth}\left(\phi \right)$$

where $f_{CT}$ indicates the chest CT images that have already been affinely aligned to the atlas images and $f_{f}$ indicates the fissures extracted from $f_{CT}$ by the automatic fissure segmentation method. $m_{CT}$ and $\;m_{f}$ represent the atlas images and the corresponding fissure images, respectively. $m_{CT}\circ \phi$, $m_{f}\circ \phi$ means the deformed CT images and fissure images. $L_{similarity}$ is the similarity measure. $L_{smooth}\left(\phi \right)$ is the regularization term of the deformation field and λ is the weight value.

(6) $$\mathrm{MSE}\left(I,\hat{I}\right)=\frac{1}{N}\overset{N}{\underset{i=1}{\sum }}{\left[I\left(i\right)-\hat{I}\left(i\right)\right]}^{2}$$

(7) $$\mathrm{Reg}\left(\phi \right)=\frac{1}{3}\left(\frac{1}{N}\sum_{i=1}^{N}G_{x}^{2}\left[\phi \left(i\right)\right]+\frac{1}{N}\sum_{i=1}^{N}G_{y}^{2}\left[\phi \left(i\right)\right]+\frac{1}{N}\sum_{i=1}^{N}G_{z}^{2}\left[\phi \left(i\right)\right]\right)$$

where $G_{x}$ expresses the image gradient along the x-axis as follows, and the expression can be extended to $G_{y}$ and $G_{z}$.

(8) $$G_{x}\left[\phi \left(i\right)\right]=\phi \left(i_{x}+1,\;i_{y},i_{z}\right)-\phi \left(i_{x},\;i_{y},i_{z}\right)$$

To avoid insufficient deformation, the warp field is not strictly forced to be flat. The abrupt changes in the deformation field lead to coarseness at the lobar border. A post-processing step is necessary, which is designed to cope with the unreasonable segmentation. The voxels near the lobar border are reassigned to the label of their closet voxel. Moreover, the result of the former lung segmentation is used to refine the lung border.

## 3. Results

### 3.1. Experiment

We compare the performance of the proposed method with the FRV-Net method published in [22]. The FRV-Net method separates the lobes by using a 3D fully convolutional neural network with regularization. The FRV-Net method was trained with 14 CT scans (consistent with the original method) randomly selected from our custom data. The training data and test data were obtained by making manual adjustments after automatic lobe segmentation using the Pulmonary Toolkit (available in a Github repository: https://github.com/tomdoel/pulmonarytoolkit, accessed on 1 September 2022). The manual lobe segmentation results were visually checked and edited by a radiologist. The CT scan data needs to be resized to 256 × 256 × 256 and then randomly sampled in 128 × 128 × 128 before input to the FRV-Net for training.

The proposed network and the FRV-Net method were implemented on an Ubuntu server with a NVIDIA Tesla V100 GPU. Training and testing are performed in Python using the Keras framework with a Tensorflow backend.

(1) Image data

The atlas with complete pulmonary lobe segmentation results was taken from the Chinese multisubject statistical human model. The atlas image size is 358 × 358 × 325 and the pixel size is 1.0 × 1.0 × 1.0 mm^3^. To test the robustness of our method, data from patients with different COPD status were used in this paper. Cases with COPD may change the morphology of the lobar borders or result in some confusing fissure-like structures. The data were scanned at the Department of Radiology, Binzhou Medical University Hospital. The information about the enrolled CT scans is summarized in Table 1. It covers different degrees of pathological data. At the same time, all the bits of data we got were manually marked.

In addition, there were 181 groups of precious data, including 171 groups for training and 10 groups for testing, and all of them were manually segmented labels.

(2) Data preprocessing

The CT test images were first affinely aligned to the atlas using the Elastix Toolbox [31] version 4.8 in Windows. In order to facilitate the registration of CT images with the atlas after affine alignment, the images after affine alignment should also be resized to the atlas image size. The detail settings could be specified in the parameter file. In the affine registration, mutual information (MI) was used to measure the similarity, and the adaptive stochastic gradient descent optimizer worked in each of the 250 iterations for a 4-level multiresolution registration strategy. With the pre-alignment, data was relocated to the atlas images’ origin and resampled to the same matrix and voxel size as the atlas images.

The values of the affinely aligned images were clamped to the range between −1000 and −200 Hounsfield units (HU) for better visualization of fissures. It allows the network to learn the relationship between the whole lung and the local fissures. After clamping, the images were linearly normalized to [0, 1].

The detected fissures are filtered by a Gaussian function. A clear, enhanced image of the pulmonary fissure was obtained by filtering. Since the registration is an optimization problem to minimize the cost function, a new image with fissures added could guide the search in the right direction.

(3) Experiments

The prediction of our proposed method was evaluated by the dice coefficient, defined in Equation (9). X and GT are the result of the lobe segmentation and the ground truth, respectively. $\cap^{\;}$ represents the intersection of two sets. | | means the number of elements in the given set.

(9) $$D\mathrm{ice}\left(X,\;\mathrm{GT}\right)=\frac{2\times \left|X\cap \mathrm{GT}\right|}{\left|X\right|+\left|\mathrm{GT}\right|}$$

In the next section, the proposed method’s performance is verified from two aspects, including visual and quantitative evaluation.

### 3.2. Results

The performance comparison of the lobe segmentation methods is provided in the form of qualitative visualization and quantitative assessment.

We chose the direct application of Voxelmorph as the baseline, and the experimental results are shown in Table 2.

**Figure 4:**
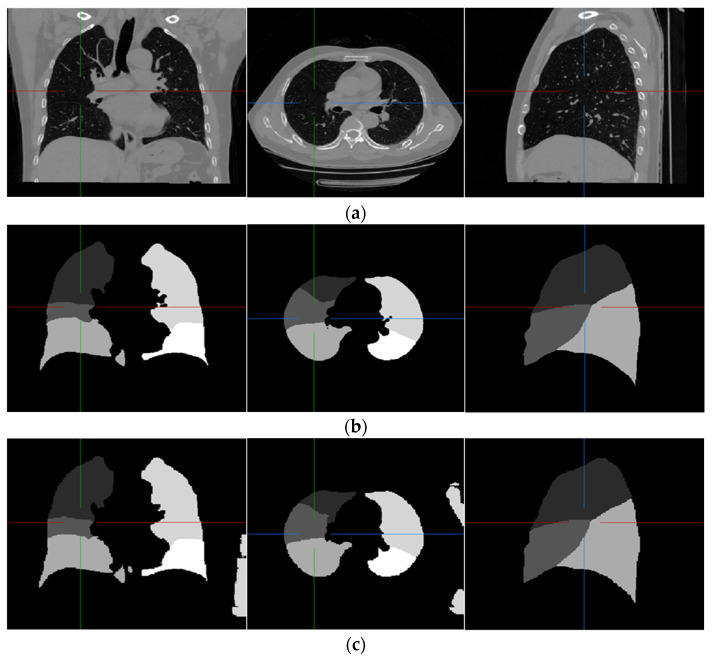

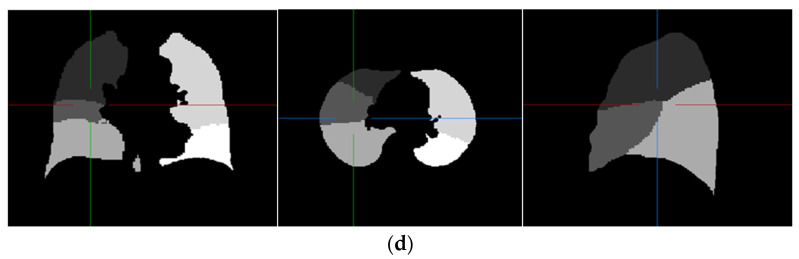
An example of the results of the experiment. (**a**) Original scan; (**b**) ground-truth; (**c**) FRV-Net; and (**d**) proposed method.

**Figure 5:**
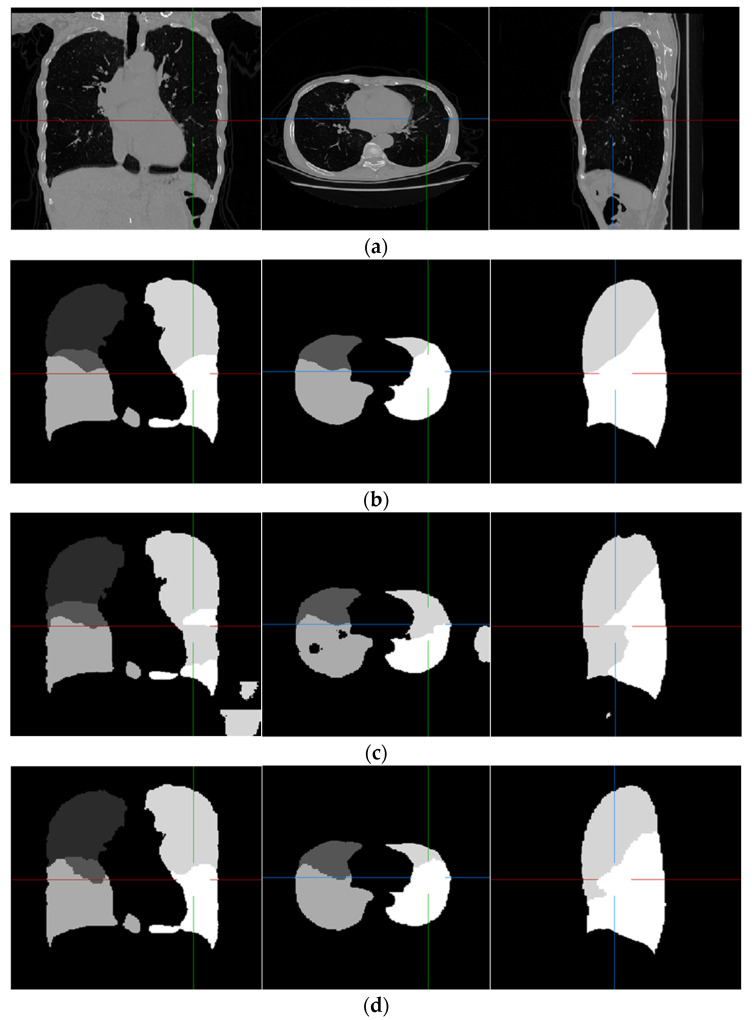
An example of the results of the experiment, which is largely different from the training dataset. (**a**) Original scan; (**b**) ground-truth; (**c**) FRV-Net; and (**d**) proposed method.

Examples of pulmonary lobe segmentation are shown in Figure 4 and Figure 5 for qualitative visualization. In Figure 4, a case with a general morphology of the lung is presented. In Figure 5, a case with morphological specificity is selected.

From Figure 4 and Figure 5, it is easy to observe that the results of the FRV-Net are interfered with by the false identification in the background. Additionally, there are irrational holes in the segmented lobe of the FRV-Net. Figure 4 shows that the proposed method could obtain a comparable segmentation result with the FRV-Net method. While in cases with large anatomical differences in the training dataset, the proposed method could avoid a wide range of misclassifications, owning to the aid of the fissures (see Figure 5).

The quantitative evaluation is expressed in Table 3. The results of the FRV-Net are further processed by masking the background voxels for a fair comparison, which is recorded as the revised FRV-Net method.

The dice coefficient of the revised FRV-Net method is significantly improved for the left upper lobe. Even compared with the revised FRV-Net method, the result of the proposed method is competitive. Furthermore, the proposed method could prevent interference from the pathological changes in morphology and avoid unreasonable holes in the segmented lobes. Generally, the proposed method could segment the pulmonary lobes accurately and robustly.

## 4. Discussion

In view of the low accuracy and instability of lung lobe segmentation in CT data with uneven image quality, this work presented an automatic lobe segmentation method that integrated the information from the fissures, the entire lung, and the prior atlas. The results show that the proposed method is valid for the segmentation of the pulmonary lobes, with an overall dice coefficient of 0.953. Compared with the FRV-Net, the proposed method outperforms it in robustness (refer to Figure 5). A shortcoming of the comparison is that test cases are limited due to the lack of manually lobe-labeled data. In further research, the effectiveness of the proposed method is expected to be verified by another annotated dataset.

The lobe segmentation approach proposed in this work has the aid of the fissure, a physical border. The success of the detection of the fissures affects the performance of the proposed segmentation method. It requires the test CT images to have a certain resolution, which allows the visualization of the fissures in computer vision. However, there will be cases in which the lung fissure cannot be detected or the detected lung fissure is incomplete. We also need to think of measures to deal with such problems. Simultaneously, the extent of the lesion-induced changes in the lung parenchyma is limited to guarantee the region-growing-based lung segmentation. Inspired by the fact that radiologists infer the incomplete fissures utilizing composite information from the surrounding structures, the airways and vascular trees will have to be taken into consideration in future studies. It would help to segment the pulmonary lobes robustly when the fissures failed to be detected.

The registration-based segmentation method could make use of the prior knowledge of the atlas. However, restricted by the unitary information of the applied atlas, the accuracy of the registration between the various lobes and the atlas lobes varied in different subjects. In further research, it is necessary to take advantage of the Chinese multisubject statistical human model. The statistical model is desired to be registered to the test CT images and deformed by adjusting the parameters of the morphology to generate a customized atlas. A congruent atlas contributes to enhancing the accuracy of lobe registration and alleviating the coarseness at the lobe border.

The input of the learning-based registration used in this work needs to be affinely aligned via the Elastix Toolbox, which makes the processing of the data complex. An end-to-end registration network, including rigid and nonrigid alignment, would attract much interest in the future. Furthermore, an automatic segmentation of the fissures using a deep learning approach also contributes to the simplification of data processing.

The atlas fissure was combined with an atlas image, and the sample fissure was combined with the affinely aligned CT image. These two sets of data are used as the input to VoxelMorph to obtain the deformation field information. Finally, the lung lobe annotation atlas is mapped to the deformation field from the registration to obtain the results of lung lobe segmentation.

## 5. Conclusions

In this paper, a learning-based registration method is presented for automatic lobe segmentation. A fissure-aided registration network is designed to strengthen the role of fissures in registering the test CT images to the prior atlas. The result of lobe segmentation is obtained by mapping the deformation field from the registration on the lobe-annotated atlas. When we add registration after fissure extraction, we can fuse the fissure and registration information, and improve the accuracy of lung lobe segmentation, so that our method has the advantages of both registration and fissure extraction. The evaluation suggests that the proposed method could perform comparably with other learning-based segmentation methods in accuracy, while outperforming them in robustness, especially in new cases that differ significantly from the training data.

## Figures and Tables

**Table 1:** Statistics of the enrolled CT scans.

	Training	Recon Matrix	Slice Thickness	Test
	GE Optima CT660 GE LightSpeed VCT GE Revolution CT GE Optima CT670	512 × 512	0.625 mm	Optima CT660 GE LightSpeed VCT
	Philips Brilliance 64	768 × 768	0.7 mm	
Ratio	90:42:18:11:10			8:2

**Table 2:** Quantitative summary of the direct application of Voxelmorph and the proposed method.

	Method		
		Voxelmorph	Proposed Method
Mean ± SD	RU	0.652 ± 0.042	0.956 ± 0.023
	RM	0.404 ± 0.042	0.904 ± 0.044
	RL	0.592 ± 0.067	0.968 ± 0.011
	LU	0.667 ± 0.035	0.971 ± 0.011
	LL	0.640 ± 0.075	0.967 ± 0.015
	Overall	0.582 ± 0.113	0.953 ± 0.035

**Table 3:** Quantitative summary of the dice coefficient for the lobe segmentation methods. RU: right upper lobe, RM: right middle lobe, RL: right lower lobe, LU: left upper lobe, LL: left lower lobe, SD: standard deviation.

		Method		
		FRV-Net	FRV-Net-Revised	Proposed Method
Mean ± SD	RU	0.973 ± 0.008	0.975 ± 0.005	0.956 ± 0.023
	RM	0.947 ± 0.021	0.948 ± 0.021	0.904 ± 0.044
	RL	0.975 ± 0.008	0.975 ± 0.008	0.968 ± 0.011
	LU	0.597 ± 0.095	0.968 ± 0.024	0.971 ± 0.011
	LL	0.962 ± 0.024	0.962 ± 0.024	0.967 ± 0.015
	Overall	0.891 ± 0.154	0.966 ± 0.021	0.953 ± 0.035

## Data Availability

The data presented in this study are inavailable.
